# Association of Clinical and Demographic Characteristics With Response to Electroconvulsive Therapy in Mania

**DOI:** 10.1001/jamanetworkopen.2022.18330

**Published:** 2022-06-23

**Authors:** Katarzyna Popiolek, Susanne Bejerot, Mikael Landén, Axel Nordenskjöld

**Affiliations:** 1 University Health Care Research Centre, Faculty of Medicine and Health, Örebro University, Örebro, Sweden; 2 Institute of Neuroscience and Physiology, Sahlgrenska Academy at Gothenburg University, Gothenburg, Sweden; 3 Department of Medical Epidemiology and Biostatistics, Karolinska Institutet, Stockholm, Sweden

## Abstract

**Importance:**

Knowledge of the effectiveness of electroconvulsive therapy (ECT) in the treatment of manic episodes is based on clinical experience, but empirical evidence is scarce. Moreover, prognostic factors associated with response to ECT in patients with mania are poorly understood.

**Objective:**

To investigate the response to ECT in patients with manic episodes.

**Design, Setting, and Participants:**

This nationwide, register-based observational cohort study was conducted using data from patients admitted to psychiatric departments in Sweden that reported data to the Swedish National Quality Registry for ECT (Q-ECT). Patients admitted to any hospital in Sweden and receiving ECT for a manic episode between 2012 and 2019 were considered for inclusion (605 individuals). The outcome, Clinical Global Impression Improvement scale (CGI-I) score, was available in 571 patients. Data from several national registers were combined to determine clinical and sociodemographic factors. Analysis of data occurred from April through September 2021.

**Exposures:**

Patients treated with ECT for a mania were identified from the Q-ECT.

**Main Outcomes and Measures:**

Response to ECT was defined by a CGI-I score of 1 (very much improved) or 2 (much improved). Remission was defined as a Clinical Global Impression Severity scale (CGI-S) score of 1 (reference range or not ill) or 2 (minimally ill) within 1 week after ECT. Univariate and multivariable regression models were used to investigate associations of sociodemographic factors, psychopharmacology, and comorbidities with response.

**Results:**

Among 571 patients with mania treated with ECT (211 [37.0%] men; median [IQR] age, 46 [31-59] years), 482 patients (84.4%) responded to ECT. Comorbid anxiety and obsessive-compulsive disorder (OCD) were associated with lower odds of response to ECT (adjusted odds ratio [aOR], 0.48; 95% CI, 0.25-0.90 and aOR, 0.17; 95% CI, 0.06-0.56, respectively). Patients who were markedly ill (aOR, 2.93; 95% CI, 1.23-7.00), severely ill (aOR, 2.60; 95% CI, 1.06-6.34), or among the most extremely ill (aOR, 7.94; 95% CI, 2.16-29.21) according to CGI-S score had higher odds of response than those with mild or moderate illness.

**Conclusions and Relevance:**

This study found that ECT was associated with improvement for mania in clinical settings, with especially high response rates in patients with severe illness and those without comorbid anxiety or OCD.

## Key Points

**Question:**

What outcomes are associated with electroconvulsive therapy (ECT) for mania, and what patient characteristics are associated with beneficial outcomes?

**Findings:**

In this cohort study of 571 patients with mania who were treated with ECT, 84% responded. Patients with more severe conditions without comorbidities of anxiety and obsessive-compulsive disorder had a significantly higher response rate.

**Meaning:**

This study found that ECT was associated with improvement for mania, with especially high response rates for patients with severe illness and no comorbidities.

## Teaser

This cohort study examines the outcomes of electroconvulsive therapy and the association of clinical and demographic characteristics with response in patients with mania.

## Introduction

Manic episodes are a psychiatric emergency. The condition confers poor judgment that places the patient or others at risk of being harmed and has potential negative social and economic consequences for patients and their environment. Achieving rapid control of symptoms is crucial, and a delay in response can have serious consequences. Guidelines recommend various pharmacological options as first-line treatments for mania^1,2,3,4,5,6^ that all include a mood stabilizer (eg, lithium or valproate), an antipsychotic, or both. However, not all patients respond to first-line treatment. The percentage of patients who showed at least moderate response to lithium was approximately 40% to 80%.^7^ A pooled analysis of studies on the outcomes associated with valproate revealed that 54% of patients experienced at least a 50% reduction in symptoms,^8,9,10^ and 40% to 73% of patients with mania achieved response to antipsychotics.^11,12,13,14,15^

Most guidelines recommend electroconvulsive therapy (ECT) as a second-line treatment for mania, and it is especially recommended for pregnant women or patients with severe mania. However, prognostic factors associated with a good response to ECT in individuals with mania have not been extensively investigated. If such factors could be identified, it may be possible to increase response rates through the use of a more personalized approach. Previous findings suggest that poor premorbid adjustment,^16,17^ psychotic features,^18^ anger, suspiciousness, and irritability^19^ were associated with a poorer response to ECT. One study^20^ reported that greater severity of manic symptoms was associated with a better response to ECT, although this association was not confirmed in the study by Mukherjee and collegues.^17^ The Swedish National Quality Registry for ECT (Q-ECT) is an opt-out register with 90% coverage^21^ and high validity.^22^ It contains detailed information on ECT administered across all ECT centers in Sweden. In this study, Q-ECT was combined with several other national registers to yield data on demographic and clinical factors as potential prognostic factors associated with response to ECT in patients with mania.

The aim of this study was to investigate response and remission rates after ECT and identify factors associated with response to ECT in patients with mania. Factors of interest were sociodemographic features, such as age, sex, education level, and cohabitation status, and clinical features, such as severity of symptoms, baseline pharmacological treatment, and psychiatric comorbidities.

## Methods

The Ethical Review Authority in Uppsala, Sweden approved this cohort study and determined that because all data were pseudonymized and individuals were not identifiable at any time, participants did not need to be informed of the study or asked to provide consent. This report followed the Strengthening the Reporting of Observational Studies in Epidemiology (STROBE) reporting guideline for observational studies.

### Design

This was a nationwide, register-based observational study. Data from several national registers were compiled for this study. Associations between selected clinical and demographical variables and response to ECT were investigated. Univariate and multivariable analyses were conducted to adjust for confounding variables.

### Participants

All individuals admitted to any hospital in Sweden and treated with ECT for mania between 2012 and 2019 according to the Q-ECT were considered for inclusion (605 individuals). Patients were diagnosed according to the *International Statistical Classification of Diseases and Related Health Problems, Tenth Revision* (*ICD-10*),^23^ which included diagnoses of F30.1 (manic episode without psychotic symptoms), F30.2 (manic episode with psychotic symptoms), F31.1 (bipolar disorder, current manic episode without psychotic features), F31.2 (bipolar disorder, current manic episode with psychotic features), and F31.6 (bipolar disorder, current mixed episode). Patients with a missing Clinical Global Impression Improvement scale (CGI-I) score were excluded, leaving 571 patients available for analysis. A description of included and excluded patients is presented in Table 1. For each individual, the first series of ECT treatments for mania during the study period was analyzed. Analyses were conducted for the entire study population, and stratified analyses were conducted for subgroups of patients with manic or mixed episodes and for men and for women separately.

**Table 1.** Sociodemographic and Clinical Characteristics of Patients and Odds of Inclusion

Characteristic	Patients, No. (%)		Odds of study inclusion	
	Study cohort (N = 571)	Excluded (n = 34)	OR (95% CI)	aOR (95% CI)
Sex				
Women	360 (63.0)	25 (70.6)	1 [Reference]	1 [Reference]
Men	211 (37.0)	10 (29.4)	1.41 (0.66-3.00)	0.84 (0.34-2.09)
Age, median (IQR), y	46 (31-59)	50 (32-65)	1.00 (0.98-1.02)	1.02 (0.99-1.05)
Education				
<High school	248 (43.4)	21 (61.8)	1 [Reference]	1 [Reference]
High school	120 (21.0)	6 (17.6)	1.69 (0.67-4.31)	1.74 (0.56-5.45)
Some college (<3 y)	79 (13.8)	3 (8.8)	2.23 (0.65-7.67)	1.37 (0.36-5.30)
College (≥3 y)	103 (18.0)	3 (8.8)	2.91 (0.85-9.96)	4.51 (1.10-18.59)
Missing data	21 (3.7)	1 (2.9)	1.78 (0.23-13.88)	0.42 (0.04-4.33)
Cohabiting				
No	311 (54.5)	21 (61.8)	1 [Reference]	1 [Reference]
Yes	252 (44.1)	13 (38.2)	1.31 (0.64-2.67)	1.91 (0.80-4.55)
Missing data	8 (1.4)	0	NA	NA
Severity of symptoms				
Mania without psychotic symptoms	183 (32.0)	22 (64.7)	1 [Reference]	1 [Reference]
Mania with psychotic symptoms	331 (58.0)	12 (35.3)	3.32 (1.60-6.86)	2.90 (1.22-6.87)
Mixed episode	57 (10.0)	0	NA	NA
Comorbidity				
Anxiety disorder	116 (20.3)	12 (35.3)	0.47 (0.23-0.97)	0.45 (0.18-1.15)
OCD	19 (3.3)	2 (5.9)	0.55 (0.12-2.47)	1.08 (0.16-7.24)
Personality disorder	79 (13.8)	6 (17.6)	0.75 (0.30-1.87)	0.97 (0.29-3.27)
ADHD	34 (6.0)	2 (5.9)	1.01 (0.23-4.41)	2.53 (0.29-22.15)
Autism	15 (2.6)	1 (2.9)	0.89 (0.11-6.95)	0.36 (0.03-4.36)
Substance abuse disorder	132 (23.1)	11 (32.4)	0.63 (0.30-1.32)	1.10 (0.42-2.88)
Type of admission				
Voluntary	141 (24.7)	16 (47.1)	1 [Reference]	1 [Reference]
Involuntary	342 (59.9))	10 (29.4)	3.88 (1.72-8.76)	7.66 (2.67-21.94)
Missing data	88 (15.4)	8 (23.5)	1.25 (0.51-3.04)	9.21 (2.25-37.76)
ECT treatment before index ECT				
No	196 (34.3)	8 (23.5)	1 [Reference]	1 [Reference]
Yes	258 (45.2)	10 (29.4)	1.05 (0.41-2.72)	1.41 (0.48-4.13)
Missing data	117 (20.5)	16 (47.1)	0.30 (0.12-0.72)	0.12 (0.04-0.40)
Pharmacotherapy within 100 d before admission				
Antidepressant	120 (21.0)	11 (32.4)	0.56 (0.26-1.17)	0.72 (0.29-1.78)
Lithium	202 (35.4)	7 (20.6)	2.11 (0.90-4.93)	4.28 (1.29-14.20)
Benzodiazepine	150 (26.3)	12 (35.3)	0.65 (0.32-1.35)	0.68 (0.27-1.70)
Antipsychotic, first generation	43 (7.5)	2 (5.9)	1.30 (0.30-5.62)	1.75 (0.34-8.91)
Antipsychotic, second generation	269 (47.1)	18 (52.9)	0.79 (0.40-1.58)	1.63 (0.43-6.28)
Anxiolytic	114 (20)	11 (32.4)	0.52 (0.25-1.10)	0.56 (0.22-1.39)
Lamotrigine	58 (10.2)	2 (5.9)	1.81 (0.42-7.74)	1.93 (0.38-9.91)
Valproate	75 (13.1)	6 (17.6)	0.71 (0.28-1.76)	1.41 (0.43-4.57)
Central stimulant	11 (1.9)	2 (5.9)	0.31 (0.07-1.48)	0.18 (0.02-1.59)
Antimanic treatment within 100 d before admission				
No	210 (36.8)	11 (32.4)	1 [Reference]	1 [Reference]
Yes	361 (63.2)	23 (67.6)	0.82 (0.39-1.72)	0.34 (0.06-1.81)

Abbreviations: ADHD, attention-deficit/hyperactivity disorder; aOR, adjusted odds ratio; ECT, electroconvulsive therapy; NA, not applicable; OCD, obsessive-compulsive disorder; OR, odds ratio.

### Data Sources

Data were retrieved from several nationwide Swedish registers and compiled by Statistics Sweden using personal identification numbers. Q-ECT provided information on age, ECT settings, presence of psychotic symptoms, compulsory care, adverse events, and assessments before, during, and after treatment. The Swedish National Patient Register is a mandatory register for all hospital admissions and doctor appointments in specialized outpatient care. It provides information on diagnoses and procedures coded according to the *ICD-10*.^24^ The Longitudinal Integrated Database for Health Insurance and Labour Market Studies (LISA) is a mandatory register for all Swedish residents aged 16 years and older. It provides detailed information on socioeconomic status, which includes family, employment, education, and income information.^25^ The Swedish Prescribed Drug Register provides information on all prescribed drugs dispensed at pharmacies in Sweden.^26^

### Variables

Response was defined as a clinician-reported CGI-I score of 1 (very much improved) or 2 (much improved) on the 7-point rating scale within 1 week after the final ECT. Remission was defined as a clinician-reported Clinical Global Impression Severity scale (CGI-S) score of 1 (reference range or not ill) or 2 (minimally ill)^27^ within 1 week of completion of ECT. Severity of mania was based on CGI-S score before ECT. Comorbid psychiatric conditions were categorized into the following groups: anxiety disorder, obsessive-compulsive disorder (OCD), attention-deficit/hyperactivity disorder, autism, personality disorder, and substance use disorder. Diagnoses according to the *ICD-10* that corresponded with categories in this study are listed in eTable 1 in the Supplement. The term *cohabiting* referred to living with a partner, regardless of marital status, or living with children. *Age of onset* referred to the first psychiatric admission. *Pharmacological treatment prior to index ECT* referred to medicines that were collected within 100 days before index admission. The following categories of medicines were considered: lithium, valproate, lamotrigine, first-generation and second-generation antipsychotics, antidepressants, benzodiazepines, anxiolytics (ie, hydroxyzine, promethazine, alimemazine, and buspirone), and central stimulants. *Antimanic treatment* referred to treatment with at least 1 of the following: lithium, valproate, and first-generation or second-generation antipsychotics. Psychopharmacological agents and corresponding Anatomical Therapeutic Chemical codes are listed in eTable 2 in the Supplement.

### ECT Device

ECT was administered using bidirectional constant-current, brief-pulse devices. These were Mecta (Mecta) or Thymatron System IV (Somatics) devices.

### Statistical Analysis

To study association between potentially prognostic variables and response to ECT, we used binary logistic regression and performed univariate and multivariable analyses. Inverse probability weighting was used to handle exclusion owing to missing information on outcome. All factors evaluated in the univariate model were used in the multivariable model: sex, age, education, cohabiting status, type of mania, comorbidities, compulsory psychiatric care, prior ECT, severity, number of treatments with ECT in the index series, and psychopharmacotherapy before ECT. The same factors were entered into a logistic regression model with remission as the dependent variable. A separate univariate analysis of association between antimanic treatment within 100 days before admission and response or remission was performed. Additionally, evaluations of factors potentially associated with outcome were performed separately for men and women (data presented in eTables 4-5 in the Supplement), as well as for mania and mixed episodes. We used 2-tailed tests of significance, and level of significance was set at *P* < .05. Statistical analyses were performed using SAS statistical software version 9.4 (SAS Institute) and SPSS statistical software version 22 (IBM). Data were analyzed from April through September 2021.

## Results

### Participants

A total of 571 patients (211 [37.0%] men; median [IQR] age, 46 [31-59] years) were included in the study. Clinical and demographic characteristics of the study population are presented in Table 1. No significant differences were found between included and 34 excluded patients regarding pharmacotherapy, comorbidities (except for anxiety), sex, or cohabiting status. Included patients had lower incidence of anxiety and higher incidence of psychotic symptoms, mixed episodes, and involuntary admission than excluded patients.

### ECT Settings

Most patients received ECT 3 times per week (the traditional frequency of such treatment in Sweden). The median (IQR; range) number of treatments in this study was 6 (4-8; 1-24) treatments. Decisions about stimulus dose and ECT series duration were made by clinicians. In accordance with national guidelines, the initial stimulus dose was usually based on patient sex, age, concomitant pharmacological treatment, and disease severity. Titration is not common in Sweden; instead, the quality of the seizure and the clinical response are evaluated continuously to adjust dosages of stimuli and anesthetics. Detailed information about ECT settings and anesthesia is presented in eTable 3 in the Supplement.

### Response and Remission Rates

**Figure 1.**
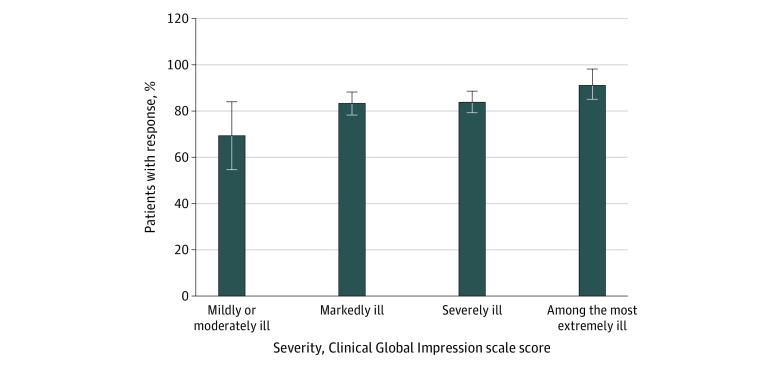
Response to Electroconvulsive Therapy by Severity of Mania Whiskers indicate 95% CIs.

Among included patients, 482 individuals (84.4%) met criteria for response to ECT. Among 89 patients who did not respond to ECT, 5 individuals (0.9%) had CGI-I scores of 5 or 6 (minimally worse or much worse) and none had CGI-I scores of 7 (very much worse). The response rates by severity of illness ranged from 69.2% (95% CI, 52.4%-83.0%) for patients who were mildly or moderately ill to 91.5% (95% CI, 82.5%-96.8%) for patients who were among the most extremely ill (Figure 1). Among 514 patients with mania, 439 individuals (85.4%) responded to ECT, and among 57 patients with mixed episodes, 43 individuals (75.4%) showed a response.

**Figure 2.**
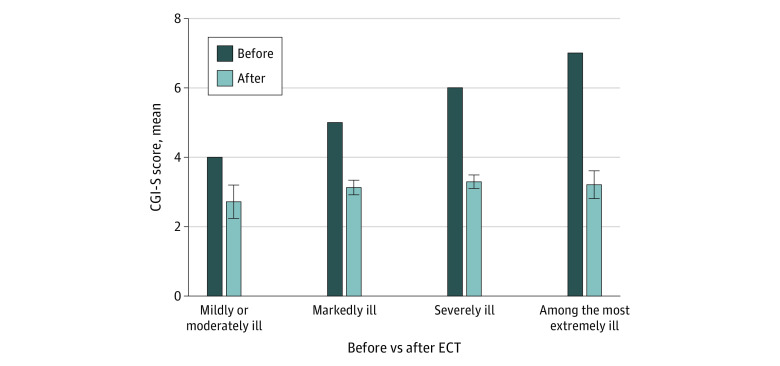
Severity Score Before and After Treatment CGI-S indicates Clinical Global Impression Severity scale; ECT, electroconvulsive therapy; whiskers, 95% CI.

Remission was achieved in 139 of 495 patients with a CGI-S score (28.1%). Remission occurred in 123 patients with mania (27.3%) and 16 patients with mixed episodes (36.4%). The differences in CGI-S score from before to after ECT are shown in Figure 2.

### Factors Associated With Response

Associations between characteristics examined in univariate and multivariable analyses and response to ECT among patients with mania are presented in Table 2. In univariate and multivariable analyses for the overall study population, comorbid anxiety (univariate: odds ratio [OR], 0.52; 95% CI, 0.31-0.87; *P* = .01; multivariable: adjusted OR [aOR], 0.48; 95% CI, 0.25-0.90; *P* = .02) and OCD (univariate: OR, 0.19; 95% CI, 0.07-0.50; *P* < .001; multivariable: aOR, 0.17; 95% CI, 0.06-0.56; *P* = .003) were associated with lower odds of responding to ECT. Severity of mania was also associated with response to ECT. For example, in multivariate analysis, patients who were markedly ill (aOR, 2.93; 95% CI, 1.23-7.00; *P* = .02), severely ill (aOR, 2.60; 95% CI, 1.06-6.34; *P* = .04), or among the most extremely ill (aOR, 7.94; 95% CI, 2.16-29.21; *P* = .002) had higher odds of responding to ECT than those with mild or moderate illness (Table 2). In univariate analyses, treatment with antidepressants was associated with lower odds of response (OR, 0.57; 95% CI, 0.34-0.96; *P* = .03), although this finding was not confirmed in multivariable analysis. There was no association between antimanic treatment within 100 days before ECT and response in univariate analysis (OR, 0.96; 95% CI, 0.60-1.54; *P* = .86).

**Table 2.** Logistic Regression of Factors Associated With Good or Very Good Response

Factor	Patients, No.	Patients with response, No.	Univariate		Multivariable	
			OR (95% CI)	*P* value	aOR (95% CI)	*P* value
Sex						
Women	360	309	1 [Reference]	NA	1 [Reference]	NA
Men	211	173	0.77 (0.48-1.22)	.26	0.60 (0.34-1.05)	.07
Age, y						
14-25	74	62	1 [Reference]	NA	1 [Reference]	NA
26-40	161	134	0.90 (0.43-1.90)	.77	0.89 (0.36-2.16)	.79
41-60	217	190	1.40 (0.67-2.94)	.37	1.52 (0.61-3.78)	.37
61-89	119	96	0.75 (0.35-1.64)	.48	0.72 (0.26-1.99)	.52
Education						
<High school	248	210	1 [Reference]	NA	1 [Reference]	NA
High school	120	99	0.84 (0.46-1.51)	.55	0.92 (0.47-1.78)	.80
Some college (<3 y)	79	71	1.63 (0.72-3.66)	.24	1.73 (0.69-4.34)	.24
College (≥3 y)	103	86	0.90 (0.48-1.68)	.74	0.96 (0.48-2.09)	.99
Missing data	21	16	0.61 (0.21-1.77)	.36	2.34 (0.39-14.01)	.35
Cohabiting						
No	311	261	1 [Reference]	NA	1 [Reference]	NA
Yes	252	217	1.22 (0.76-1.96)	.41	1.30 (0.77-2.19)	.32
Missing data	8	4	0.20 (0.05-0.81)	.02	0.05 (0.01-0.49)	.01
Type of mania						
Mania without psychosis	183	155	1 [Reference]	NA	1 [Reference]	NA
Mania with psychosis	331	284	1.14 (0.68-1.90)	.63	0.88 (0.46-1.70)	.71
Mixed episode	57	43	0.57 (0.28-1.19)	.13	0.46 (0.19-1.10)	.08
Substance abuse disorder						
No	439	373	1 [Reference]	NA	1 [Reference]	NA
Yes	132	109	0.87 (0.52-1.47)	.60	0.95 (0.51-1.77)	.87
Anxiety disorder						
No	455	393	1 [Reference]	NA	1 [Reference]	NA
Yes	116	89	0.52 (0.31-0.87)	.01	0.48 (0.25-0.90)	.02
Personality disorder						
No	492	416	1 [Reference]	NA	1 [Reference]	NA
Yes	79	66	0.90 (0.47-1.73)	.75	1.52 (0.71-3.28)	.28
OCD						
No	552	471	1 [Reference]	NA	1 [Reference]	NA
Yes	19	11	0.19 (0.07-0.50)	<.001	0.17 (0.06-0.56)	.003
ADHD						
No	537	454	1 [Reference]	NA	1 [Reference]	NA
Yes	34	28	0.88 (0.35-2.20)	.79	1.79 (0.51-6.26)	.36
Autism						
No	556	469	1 [Reference]	NA	1 [Reference]	NA
Yes	15	13	1.23 (0.27-5.53)	.79	1.14 (0.20-6.57)	.89
Compulsory psychiatric care						
No	141	115	1 [Reference]	NA	1 [Reference]	NA
Yes	324	297	1.45 (0.85-2.48)	.17	0.84 (0.42-1.68)	.62
Missing data	88	70	0.81 (0.41-1.61)	.56	0.48 (0.18-1.29)	.15
ECT treatment before index ECT						
No	196	165	1 [Reference]	NA	1 [Reference]	NA
Yes	258	218	1.02 (0.61-1.71)	.93	0.96 (0.54-1.70)	.89
Missing data	117	99	1.01 (0.53-1.93)	.97	2.13 (0.85-5.37)	.11
Severity by CGI-S score						
Mildly to moderately ill	39	27	1 [Reference]	NA	1 [Reference]	NA
Markedly ill	181	151	2.16 (0.98-4.76)	.56	2.93 (1.23-7.00)	.02
Severely ill	251	212	2.29 (1.07-4.90)	.03	2.60 (1.06-6.34)	.04
Among the most extremely ill	71	65	4.64 (1.58-13.64)	.005	7.94 (2.16-29.21)	.002
Missing data	29	27	6.25 (1.28-30.66)	.02	10.62 (1.69-66.87)	.01
ECT treatments in index series, No.						
1-5	164	128	1 [Reference]	NA	1 [Reference]	NA
6-9	305	266	2.06 (1.25-3.42)	.005	2.12 (1.21-3.70)	.008
>9	102	88	1.81 (0.92-3.58)	.09	2.25 (1.01-5.02)	.05
Age of onset, y						
<21	132	111	1 [Reference]	NA	1 [Reference]	NA
<26	111	97	1.29 (0.62-2.68)	.50	1.36 (0.59-3.14)	.47
<36	134	112	0.94 (0.50-1.81)	.85	1.21 (0.52-2.80)	.66
>35	110	93	0.97 (0.48-1.98)	.94	1.39 (0.57-3.43)	.47
Missing data	84	69	0.90 (0.43-1.87)	.78	1.02 (0.44-2.38)	.97
Psychopharmacotherapy before index admission						
Lithium						
No	369	312	1 [Reference]	NA	1 [Reference]	NA
Yes	202	170	0.98 (0.61-1.58)	.94	1.13 (0.66-1.96)	.66
Lamotrigine						
No	513	437	1 [Reference]	NA	1 [Reference]	NA
Yes	58	45	0.62 (0.32-1.20)	.16	0.66 (0.30-1.43)	.29
Antipsychotic, first generation						
No	528	448	1 [Reference]	NA	1 [Reference]	NA
Yes	43	34	0.68 (0.31-1.47)	.32	0.47 (0.19-1.17)	.11
Antipsychotic, second generation						
No	302	259	1 [Reference]	NA	1 [Reference]	NA
Yes	269	223	0.77 (0.49-1.21)	.26	0.70 (0.41-1.22)	.21
Valproate						
No	496	418	1 [Reference]	NA	1 [Reference]	NA
Yes	75	64	1.08 (0.54-2.16)	.82	1.00 (0.45-2.26)	.99
Benzodiazepine						
No	421	358	1 [Reference]	NA	1 [Reference]	NA
Yes	150	124	0.80 (0.48-1.34)	.40	0.99 (0.54-1.978)	.96
Antidepressant						
No	451	388	1 [Reference]	NA	1 [Reference]	NA
Yes	120	94	0.57 (0.34-0.96)	.03	0.71 (0.38-1.33)	.29
Anxiolytic						
No	457	382	1 [Reference]	NA	1 [Reference]	NA
Yes	114	100	1.41 (0.76-2.61)	.28	1.54 (0.79-3.02)	.21
Central stimulant						
No	560	474	1 [Reference]	NA	1 [Reference]	NA
Yes	11	8	0.56 (0.15-2.18)	.41	0.53 (0.10-2.80)	.46

Abbreviations: ADHD, attention-deficit/hyperactivity disorder; aOR, adjusted odds ratio; CGI-S, Clinical Global Impression Severity scale; ECT, electroconvulsive therapy; NA, not applicable; OCD, obsessive-compulsive disorder; OR, odds ratio.

A separate analysis was conducted among patients with mania excluding those with a mixed episode. In this analysis, men had lower odds of response to ECT than women (aOR, 0.54; 95% CI, 0.30-0.95; *P* = .034).

### ECT Settings and Response to ECT

We examined the following ECT settings: charge, pulse width, and seizure duration. No settings were found to be associated with response to ECT.

### Factors Associated With Remission

Treatment with antidepressants was associated with lower odds of achieving remission in univariate analysis (univariate: OR, 0.45; 95% CI, 0.26-0.80; *P* = .006; multivariable: aOR, 0.61; 95% CI, 0.33-1.11; *P* = .10). Cohabiting was associated with higher odds of achieving remission in univariate (OR, 1.67; 95% CI, 1.12-2.49; *P* = .01) but not multivariable analysis (aOR, 1.57; 95% CI, 1.00-2.48; *P* = .05).

## Discussion

In this cohort study, we investigated response and factors associated with response when ECT was used to treat manic episodes in 571 patients. Our main findings were that nearly 85% of patients treated with ECT for mania responded and 28% of patients achieved remission. These findings suggest that ECT may be a highly effective option for treating mania, which is in line with the literature reporting response rates of 56% to 100%.^28^ We also found that severity of mania was the factor associated with the greatest increase in odds of response to ECT.

Although few randomized studies have compared efficacy between ECT and psychopharmacological methods, 3 randomized clinical trials found that ECT was more effective than alternative treatments.^20,29,30^ Sikdar et al^30^ found that 12 of 15 patients with mania treated with ECT and chlorpromazine recovered, whereas 1 of 15 patients treated with sham ECT and chlorpromazine achieved recovery. Small et al^20^ compared ECT (17 individuals) with lithium (17 individuals) and found that 95% of patients treated with ECT improved compared with 81% of patients in the lithium group. Moreover, Mukherjee et al^29^ found that lithium in combination with antipsychotics was less effective than ECT combined with antipsychotics. In that study, 13 of 22 patients who received ECT improved, whereas none of 5 patients treated with lithium improved.

The potential association of ECT with higher rates of improvement compared with pharmacotherapy was also supported by several retrospective studies. ECT was associated with superior outcomes compared with chlorpromazine in an early study by McCabe.^31^ In this study, all 28 patients responded to ECT, whereas 18 of 28 patients responded to chlorpromazine. Furthermore, Black et al^18^ published a retrospective medical record review of 438 individuals treated over 12 years; in that study, ECT was associated with better outcomes than adequate lithium treatment. Marked improvement was achieved by 29 of 37 patients treated with ECT (78%) and 126 of 203 patients who received adequate lithium treatment (62%). Moreover, almost 70% of patients treated with lithium who failed to respond reached marked improvement if they were offered subsequent ECT treatment. However, studies with head-to-head comparisons of ECT and psychopharmacological treatments are needed to fully confirm these findings.

In our study, greater severity of mania was associated with higher odds of response to ECT. Among patients assessed as mildly and moderately ill, 69% responded, whereas the response rate among patients assessed as among the most extremely ill was 91%. Such differences in response rates by illness severity may be associated with regression to the mean, in which patients with more severe illness had greater potential to improve. However, in the analysis of CGI-S scores before and after ECT, patients who were more severely ill had greater benefits than individuals with milder disease. The value of baseline symptom severity in estimating ECT response was investigated in 2 prospective studies: the Indiana study^20^ (ECT vs lithium) and New York study^17^ (ECT vs lithium + haloperidol). Results of the Indiana study were in line with our findings, which suggests that individuals with more severe mania may have a greater likelihood of responding to ECT, while the New York study found no such association. However, the notion of symptom severity as a prognostic factor associated with response to ECT is not unique to mania. A meta-analysis by van Diermen et al^32^ found that in patients treated with ECT who had depression, baseline symptom severity was a prognostic factor associated with a better response. In addition, increased symptom severity was found to be associated with better response rates in a study of postpartum depression and psychosis.^33^

A more complex psychiatric condition, reflected by comorbid anxiety and OCD, was associated with a lower response rate in our study. This finding is in agreement with those of a case-control study by Black et al,^34^ which compared patients with complicated mania (57 individuals) and uncomplicated mania (114 individuals). Patients in the former group had a comorbid psychiatric disorder or serious medical illness in addition to mania. Individuals were then divided into different treatment groups. Overall, recovery at discharge was achieved by 68.4% of patients with uncomplicated mania compared with 45.6% of patients with complicated mania. Comorbid OCD and personality syndrome were also associated with poorer outcomes in a large cohort of patients with ECT-treated bipolar depression.^35^ Response was achieved by 80% of the total study population; however, 55% of patients with comorbid OCD achieved a response. In contrast to these findings, a study including 41 patients with mania and mixed episodes^36^ found no association between psychiatric comorbidities and ECT response. Although psychiatric comorbidities with bipolar disorder are common, Parker^37^ found that two-thirds of patients with bipolar disorder had psychiatric comorbidities. However, the nature of this association is still poorly understood.

We found that treatment with antidepressants was associated with lower odds of responding to ECT in the univariate but not multivariable analysis. No other psychopharmacotherapies were associated with higher or lower odds of response to ECT. It is worth highlighting that 63% of patients in our study were treated with 1 or more antimanic agents before admission, suggesting that these treatments may not have been sufficient in reducing symptoms of mania.

Although ECT was associated with positive outcomes in treating symptoms of mania, it can be problematic to deliver the treatment in patients with mania. Many such patients have limited insight into their disorder or the need for treatment. To deliver treatment in an ethical manner to nonconsenting or ambivalent patients with mania requires great care and close cooperation among the psychiatrist in the ward, the psychiatrist in charge of ECT, and the anesthesiologist to minimize the need for force or restraints. Moreover, the legal basis for providing ECT to patients not able to provide consent may differ by country. The Swedish act on psychiatric compulsory care leaves great room for the judgement of the psychiatrist to balance the need for safe and rapid symptom relief against the risk of a breach of integrity associated with treating patients without consent. To balance the scales, providing effective treatment for severe mania with ECT may limit the need for prolonged hospitalization, adverse effects from excessive sedative medication and repeated injections, and risk for reckless involvement in activities that characterize the manic state.

### Limitations

This study has several limitations. Because this was a nonrandomized study, associations between potential prognostic factors and treatment outcomes do not indicate causality between study variables. Another limitation is that the evaluation of response and remission was not performed using mania-specific scales. Additionally, information on pharmacological treatment after admission was not available. According to Swedish national guidelines for the treatment of mania, ECT is recommended as a second-line treatment after pharmacological treatment failure. In this study, whether patients were viewed as nonresponders or partial responders to pharmacological treatment before ECT was unknown.

## Conclusions

This study offers evidence suggesting the association of ECT with high levels of improvement in the treatment of mania in a clinical setting. Patients with the highest aORs for response were those with more severe mania, whereas patients with comorbid anxiety and OCD had lower odds of response.

## Notes

Supplement. **eTable 1.** Comorbidities **eTable 2.** Categories of Psychopharmacological Agents and Anatomical Therapeutic Chemical (ATC) Codes **eTable 3.** Electroconvulsive Therapy (ECT) Settings **eTable 4.** Logistic Regression Analysis of Factors Associated With Good or Very Good Response in Women **eTable 5.** Logistic Regression of Factors Associated With Good or Very Good Response in Men
