# Supplementary material for: Association of Clinical and Demographic Characteristics With Response to Electroconvulsive Therapy in Mania
Source: JAMA Netw Open. 2022 Jun 23;5(6):e2218330. doi: 10.1001/jamanetworkopen.2022.18330 (PMC9227004; doi:10.1001/jamanetworkopen.2022.18330)
Supplement: Supplement. — eTable 1. Comorbidities eTable 2. Categories of Psychopharmacological Agents and Anatomical Therapeutic Chemical (ATC) Codes eTable 3. Electroconvulsive Therapy (ECT) Settings eTable 4. Logistic Regression Analysis of Factors Associated With Good or Very Good Response in Women eTable 5. Logistic Regression of Factors Associated With Good or Very Good Response in Men [file jamanetwopen-e2218330-s001.pdf]

Supplemental Online Content

Popiolek K, Bejerot S, Landén M, Nordenskjöld A. Association of clinical and demographic characteristics with response to electroconvulsive therapy in mania. *JAMA Netw Open*. 2022;5(6):e2218330. doi:10.1001/jamanetworkopen.2022.18330

eTable 1. Comorbidities

eTable 2. Categories of Psychopharmacological Agents and Anatomical Therapeutic Chemical (ATC) Codes

eTable 3. Electroconvulsive Therapy (ECT) Settings

eTable 4. Logistic Regression Analysis of Factors Associated With Good or Very Good Response in Women

eTable 5. Logistic Regression of Factors Associated With Good or Very Good Response in Men

This supplemental material has been provided by the authors to give readers additional information about their work.

© 2022 Popiolek K et al. *JAMA Network Open.*

eTable 1. Comorbidities

Comorbidity category in the present study	Diagnoses according to ICD-10
Anxiety disorder	F41.0 Panic disorder [episodic paroxysmal anxiety]<br>F41.1 Generalized anxiety disorder<br>F41.2 Mixed anxiety and depressive disorder<br>F41.3 Other mixed anxiety disorders<br>F41.8 Other specified anxiety disorders<br>F41.9 Anxiety disorder, unspecified
Obsessive-compulsive disorder	F42.0 Predominantly obsessional thoughts or ruminations<br>F42.1 Predominantly compulsive acts [obsessional rituals]<br>F42.8 Other obsessive-compulsive disorders<br>F42.9 Obsessive-compulsive disorder, unspecified
Attention-deficit hyperactivity disorder (ADHD)	F90.0 Disturbance of activity and attention
Autism	F84.1 Atypical autism<br>F84.5 Asperger syndrome
Personality disorder	F60.0 Paranoid personality disorder<br>F60.1 Schizoid personality disorder<br>F60.2 Dissocial personality disorder<br>F60.3 Emotionally unstable personality disorder<br>F60.4 Histrionic personality disorder<br>F60.5 Anankastic personality disorder<br>F60.6 Anxious [avoidant] personality disorder<br>F60.7 Dependent personality disorder<br>F60.8 Other specific personality disorders<br>F60.9 Personality disorder, unspecified
Substance use disorder	F10 Mental and behavioral disorders due to use of alcohol<br>F11 Mental and behavioral disorders due to use of opioids<br>F12 Mental and behavioral disorders due to use of cannabinoids<br>F13 Mental and behavioral disorders due to use of sedatives or hypnotics<br>F14 Mental and behavioral disorders due to use of cocaine<br>F16 Mental and behavioral disorders due to use of hallucinogens<br>F18 Mental and behavioral disorders due to use of volatile solvents<br>F19 Mental and behavioral disorders due to multiple drug use and use of other psychoactive substances

© 2022 Popiolek K et al. *JAMA Network Open.*

eTable 2. Categories of Psychopharmacological Agents and Anatomical Therapeutic Chemical (ATC) Codes

Category used in this study	Pharmacological agents and corresponding ATC codes
Lithium	Lithium N05AN01
Valproate	Valproate N03AG01
Lamotrigine	Lamotrigine N03AX09
First generation antipsychotics	Haloperidol N05AD01<br>Zuklopentixol N05AF05<br>Flupentixol N05AF01<br>Perphenazine N05AB03
Second generation antipsychotics	Clozapine N05AH02<br>Risperidone N05AX08<br>Olanzapine N05AH03<br>Ziprasidone N05AE04<br>Quetiapine N05AH04<br>Aripiprazole N05AX12<br>Paliperidone N05AX13<br>Lurasidone N05AE05
Antidepressants	Citalopram N06AB04<br>Escitalopram N06AB10<br>Sertraline N06AB06<br>Paroxetine N06AB05<br>Fluoxetine N06AB03<br>Fluvoxamine N06AB08<br>Mirtazapine N06AX11<br>Reboxetine N06AX18<br>Agomelatine N06AX22<br>Bupropion N06AX12<br>Vortioxetine N06AX26<br>Venlafaxine N06AX16<br>Duloxetine N06AX21<br>Clomipramine N06AA04<br>Amitriptyline N06AA09<br>Nortriptyline N06AA10<br>Mianserin N06AX03
Benzodiazepines	Oxazepam N05BA04<br>Alprazolam N05BA12<br>Lorazepam N05BA06<br>Nitrazepam N05CD02<br>Diazepam N05BA01<br>Klonazepam N03AE01<br>Flunitrazepam N05CD03
Anxiolytics	Hydroxysine N05BB01<br>Promethazine R06AD02<br>Alimemazine R06AD01<br>Buspiron N05BE01
Central stimulants	Metylfenidat N06BA04<br>Dexamfetamin N06BA02<br>Lisdexamfetamin N06BA12

© 2022 Popiolek K et al. *JAMA Network Open.*

eTable 3. Electroconvulsive Therapy (ECT) Settings

		First ECT in the index series	Last ECT in the index series
Electrode placement (%)	Unilateral	372 (65%)	393 (69%)
	Bitemporal	190 (33%)	166 (29%)
	bifrontal	8 (1%)	8 (1%)
Poulse width (mean)		0.5 ms (SD, 0.14 ms)	0.55 ms (S, 0.17 ms)
Frequency (mean)		62 Hz (SD, 20 Hz)	68 Hz (SD, 20.6 Hz)
Duration (mean)		6.7 s (SD,1.3 s)	6.9 s (SD, 1.2 s)
Current (mean)		840 mA (SD, 51 mA)	842 mA (SD, 51 mA)
Charge (mean)		355 mC (SD, 161 mC)	429 mC (SD, 198 mC)
Seizure duration according to EEG (mean)		44 s (SD, 24 s)	37 s (SD, 18 s)
Anaesthesia (n, %)	Thiopental	311 (54.5)	
	Propofol	174 (30.5%)	
	Remifentanil	8 (1.4%)	
	Ketamin	1 (0.2%)	
	Other	4 (0.8%)	

Information on ECT settings was missing for 53–73 (12–16%) individuals.

© 2022 Popiolek K et al. *JAMA Network Open.*

eTable 4. Logistic Regression Analysis of Factors Associated With Good or Very Good Response in Women

			Univariate		Multivariable	
		n	OR (95% CI)	p-value	OR (95% CI)	*p*-value
Age, years	14–25	33	Reference			
	26–40	113	1.63 (0.61-4.38)	0.33	2.62 (0.78-8.76)	0.12
	41–60	134	1.99 (0.74-5.31)	0.17	2.80 (0.81-9.67)	0.10
	61–89	80	1.53 (0.54-4.30)	0.42	1.63 (0.41-6.46)	0.49
Education	Less than high school	159	Reference			
	High school	64	0.88 (0.38-2.05)	0.77	0.82 (0.32-2.12)	0.67
	Some college < 3 years	51	1.08 (0.41-2.85)	0.88	1.06 (0.36-3.13)	0.92
	College ≥ 3 years	74	0.68 (0.32-1.44)	0.31	0.51 (0.21-1.23)	0.14
	Missing	12	0.43 (0.11-1.73)	0.24	0.95 (0.14-6.26)	0.96
Cohabiting	No	192	Reference			
	Yes	165	1.06 (0.58-1.95)	0.84	1.22 (0.56-2.63)	0.62
	Missing	3	**0.08 (0.01-0.93)**	**0.04**	**0.01 (0.00-1.06)**	**0.05**
Type of mania	Mania without psychosis	119	Reference			
	Mania with psychosis	199	1.05 (0.53-2.10)	0.89	0.94 (0.36-2.45)	0.90
	Mixed episode	42	**0.36 (0.15-0.85)**	**0.02**	0.50 (0.18-1.36)	0.17
Substance abuse disorder	No	291	Reference			
	Yes	69	0.74 (0.36-1.49)	0.39	0.73 (0.31-1.71)	0.47
Anxiety disorder	No	274	Reference			
	Yes	86	**0.38 (0.20-0.71)**	**0.002**	**0.46 (0.20-1.05)**	**0.06**
Personality disorder	No	300	Reference			
	Yes	60	0.60 (0.29-1.22)	0.16	0.93 (0.42-2.10)	0.87
OCD	No	347	Reference			
	Yes	13	**0.17 (0.06-0.54)**	**0.003**	**0.09 (0.02-0.31)**	**0.001**
ADHD	No	339	Reference			
	Yes	21	0.68 (0.22-2.12)	0.51	1.39 0.25-7.65)	0.71
Autism	No	350	Reference			
	Yes	10	0.65 (0.13-3.16)	0.59	0.47 (0.06-3.80)	0.48
Compulsory psychiatric care	No	103	Reference			
	Yes	202	1.62 (0.82-3.20)	0.17	0.90 (0.37-2.21)	0.82
	Missing	55	0.71 (0.31-1.62)	0.41	0.43 (0.12-1.58)	0.20
	No	118	Reference			

© 2022 Popiolek K et al. *JAMA Network Open.*

ECT treatment prior to index ECT	Yes	168	0.92 (0.46-1.85)	0.81	1.12 (0.52-2.45)	0.77
	Missing	74	0.68 (0.31-1.53)	0.36	1.29 (0.32-5.13)	0.72
Severity according to CGI-S	Mildly–moderately ill	30	Reference			
	Markedly ill	115	1.45 (0.55-3.83)	0.46	1.25 (0.43-3.61)	0.68
	Severely ill	165	2.09 (0.80-5.46)	0.13	1.77 (0.56-5.60)	0.33
	Among the most extremely ill	34	4.87 (0.93-25.62)	0.06	7.13 (0.66-77.61)	0.11
	Missing	16	4.57 (0.51-40.92)	0.18	**41.43 (1.56-1097.99)**	**0.03**
Number of ECT in the index series	1-5	105	Reference			
	6-9	190	1.43 (0.74-2.78)	0.29	1.57 (0.71-3.48)	0.27
	>9	65	1.29 (0.54-3.07)	0.57	1.46 (0.55-3.83)	0.45
Age of onset	<21	78	Reference			
	<26	63	1.60 (0.60-4.29)	0.35	0.90 (0.27-3.03)	0.86
	<36	103	1.09 (0.49-2.42)	0.84	0.80 (0.31-2.04)	0.64
	>35	66	1.69 (0.63-4.51)	0.30	1.52 (0.47-4.89)	0.48
	Missing	50	1.05 (0.40-2.75)	0.92	0.94 (0.28-3.15)	0.92
Psychopharmacotherapy prior to index admission						
Lithium	No	232	Reference			
	Yes	128	0.76 (0.41-1.39)	0.37	0.82 (0.43-1.60)	0.57
Lamotrigine	No	318	Reference			
	Yes	42	0.56 (0.25-1.25)	0.16	0.71 (0.29-1.72)	0.45
Antipsychotics, first generation	No	328	Reference			
	Yes	42	0.88 (0.32-2.40)	0.80	0.71 (0.29-1.72)	0.45
Antipsychotics, second generation	No	180	Reference			
	Yes	180	0.66 (0.36-1.20)	0.18	0.85 (0.42-1.73)	0.66
Valproate	No	312	Reference			
	Yes	48	0.96 (0.41-2.28)	0.93	0.93 (0.36-2.40)	0.89
Benzodiazepines	No	247	Reference			
	Yes	113	0.67 (0.36-1.23)	0.20	0.80 (0.38-1.66)	0.54
Antidepressants	No	278	Reference			
	Yes	82	0.66 (0.34-1.29)	0.23	0.79 (0.35-1.80)	0.58
Anxiolytics	No	271	Reference			
	Yes	89	1.08 (0.54-2.17)	0.83	1.16 (0.54-2.52)	0.70
Central stimulants	No	353	Reference			
	Yes	7	0.99 (0.18-8.40)	0.99	1.02 (0.08-12.95)	0.99

© 2022 Popiolek K et al. *JAMA Network Open.*

ADHD, attention-deficit hyperactivity disorder; CGI-S, Clinical Global Assessment Severity Scale; CI, confidence interval; ECT, electroconvulsive therapy; OCD, obsessive-compulsive disorder; OR, odds ratio; SD, standard deviation.

© 2022 Popiolek K et al. *JAMA Network Open.*

eTable 5. Logistic Regression of Factors Associated With Good or Very Good Response in Men

			Univariate		Multivariable	
		n	OR (95% CI)	p-value	OR (95% CI)	*p*-value
Age, years	14–25	41	Reference			
	26–40	48	0.47 (0.15-1.48)	0.20	0.18 (0.03-1.15)	0.07
	41–60	83	0.91 (0.29-2.82)	0.87	0.71 (0.11-4.48)	0.72
	61–89	39	0.35 (0.11-1.14)	0.08	**0.07 (0.01-0.71)**	**0.03**
Education	Less than high school	89	Reference			
	High school	56	0.93 (0.41-2.11)	0.86	2.48 (0.82-7.54)	0.11
	Some college < 3 years	28	3.30 (0.72-15.20)	0.13	**8.63 (1.48-50.35)**	**0.02**
	College ≥ 3 years	29	1.59 (0.49-5.13)	0.44	3.80 (0.66-21.94)	0.14
	Missing	9	0.89 (0.17-4.64)	0.89	-	
Cohabiting	No	119	Reference			
	Yes	87	1.37 (0.65-2.87)	0.41	1.80 (0.46-7.14)	0.40
	Missing	5	0.36 (0.06-2.28)	0.28	**-**	
Type of mania	Mania without psychosis	64	Reference			
	Mania with psychosis	132	1.21 (0.57-2.58)	0.63	0.73 (0.22-2.44)	0.61
	Mixed episode	15	1.66 (0.33-8.28)	0.54	0.66 (0.09-4.92)	0.68
Substance abuse disorder	No	148	Reference			
	Yes	63	1.06 (0.49-2.28)	0.89	0.86 (0.28-2.64)	0.80
Anxiety disorder	No	181	Reference			
	Yes	30	**0.86 (0.33-2.27)**	**0.76**	**0.32 (0.08-1.36)**	**0.12**
Personality disorder	No	192	Reference			
	Yes	19	4.30 (0.56-33.22)	0.16	191.69 (13.76-1270.71)	<0.001
OCD	No	205	Reference			
	Yes	6	**0.43 (0.08-2.42)**	**0.34**	**0.44 (0.04-4.31)**	**0.48**
ADHD	No	198	Reference			
	Yes	13	1.22 (0.26-5.76)	0.80	4.03 (0.54-30.09)	0.17
Autism	No	206	Reference			
	Yes	5*	-	-	-	-
Compulsory psychiatric care	No	38	Reference			
	Yes	140	1.58 (0.66-3.77)	0.30	1.09 (0.20-6.03)	0.92
	Missing	33	1.40 (0.44-4.45)	0.57	0.77 (0.10-5.93)	0.80
	No	78	Reference			

© 2022 Popiolek K et al. *JAMA Network Open.*

ECT treatment prior to index ECT	Yes	90	1.11 (0.52-2.37)	0.79	0.49 (0.16-1.47)	0.20
	Missing	43	1.96 (0.66-5.79)	0.22	9.10 (1.53-54.17)	0.02
Severity according to CGI-S	Mildly–moderately ill	9	Reference			
	Markedly ill	66	**7.00 (1.60-30.66)**	**0.01**	256.00 (24.30-2697.11)	<0.001
	Severely ill	86	**4.72 (1.15-19.41)**	**0.03**	**80.06 (7.68-834.56)**	**<0.001**
	Among the most extremely ill	37	**10.31 (1.93-55.05)**	**0.006**	396.17 (27.93-5620.34)	<0.001
	Missing	13	15.00 (1.33-169.87)	0.03	288.95 (9.47-8815.21)	0.001
Number of ECT in the index series	1-5	59	Reference			
	6-9	115	2.93 (1.35-6.36)	0.007	**4.67 (1.54-14.18)**	**0.007**
	>9	37	2.81 (0.94-8.39)	0.06	**11.43 (1.10-119.07)**	**0.04**
Age of onset	<21	54	Reference			
	<26	48	1.02 (0.34-3.06)	0.97	1.25 (0.29-5.33)	0.76
	<36	31	0.73 (0.23-2.32)	0.59	1.28 (0.23-7.16)	0.78
	>35	44	0.59 (0.21-1.66)	0.32	2.11 (0.25-17.81)	0.49
	Missing	34	0.67 (0.22-2.06)	0.49	0.32 (0.07-1.44)	0.14
Psychopharmacotherapy prior to index admission						
Lithium	No	137	Reference			
	Yes	74	1.41 (0.65-3.03)	0.38	1.92 (0.49-7.60)	0.35
Lamotrigine	No	195	Reference			
	Yes	16	0.63 (0.19-2.08)	0.45	0.74 (0.05-11.10)	0.83
Antipsychotics, first generation	No	200	Reference			
	Yes	11	0.36 (0.10-1.29)	0.12	0.07 (0.01-0.88)	0.04
Antipsychotics, second generation	No	122	Reference			
	Yes	89	1.00 (0.49-2.04)	0.99	0.50 (0.17-1.48)	0.21
Valproate	No	184	Reference			
	Yes	27	1.30 (0.42-4.02)	0.64	0.44 (0.07-2.84)	0.39
Benzodiazepines	No	174	Reference			
	Yes	37	1.17 (0.45-3.02)	0.76	1.10 (0.26-4.72)	0.90
Antidepressants	No	173	Reference			
	Yes	38	**0.45 (0.20-1.02)**	**0.06**	0.77 (0.19-3.13)	0.72
Anxiolytics	No	186	Reference			
	Yes	25	2.76 (0.62-12.25)	0.18	4.34 (0.89-21.19)	0.07
	No	207	Reference			

© 2022 Popiolek K et al. *JAMA Network Open.*

Central stimulants	Yes	4	0.21 (0.03-1.54)	0.13	0.05 (0.00-1.70)	0.09

ADHD, attention-deficit hyperactivity disorder; CGI-S, Clinical Global Assessment Severity Scale; CI, confidence interval; ECT, electroconvulsive therapy; OCD, obsessive-compulsive disorder; OR, odds ratio; SD, standard deviation.

*all 5 patients with autism responded to ECT

© 2022 Popiolek K et al. *JAMA Network Open.*
